# Diagnosis and Management of Ventricular Septal Defects

**DOI:** 10.31083/j.rcm2511411

**Published:** 2024-11-20

**Authors:** P. Syamasundar Rao

**Affiliations:** ^1^Children’s Heart Institute, UT Health McGovern Medical School, Houston, TX 77030, USA

**Keywords:** ventricular septal defects, echocardiography, surgical closure, transcatheter occlusion

## Abstract

This review addresses the diagnosis and management of ventricular septal defects (VSDs). The VSDs are classified on the basis of their size, their number, and their location in the ventricular septum. Natural history of VSDs includes spontaneous closure, development of pulmonary hypertension, onset of infundibular obstruction, and progression to aortic insufficiency. While initial diagnostic approaches such as careful history-taking, physical examination, chest X-rays, and electrocardiograms provide basic information, echo-Doppler studies are essential for assessing the defect's clinical significance and determining the need for intervention. Intervention is usually indicated for symptomatic patients with moderate- to large-sized VSDs. Surgical closure is advised for perimembranous, supracristal and inlet VSDs, and for deficits involving prolapsed aortic valve leaflets. While percutaneous methods to occlude perimembranous VSDs with Amplatzer Membranous VSD Occluder are feasible, they are not recommended due to a notable risk of inducing complete heart block in a significant number of patients. Alternatively, percutaneous and hybrid methods employing the Amplatzer Muscular VSD Occluder are effective for treating large muscular VSDs. The majority of treatment options have demonstrated satisfactory outcomes. However, practitioners are urged to exercise caution in managing small defects to avoid unnecessary procedures and to ensure timely intervention for large VSDs to prevent pulmonary vascular obstructive disease.

## 1. Introduction

Ventricular septal defects (VSDs) are the most common congenital heart defects (CHDs), surpassed only by the bicuspid aortic valve, and accounts for 20 to 25% of all CHDs [1, 2, 3]. The CHDs themselves are present in 0.8% of live births [1]. This review will present a classification system, natural history, detail both clinical and non-invasive features, and explore indications for intervention through medical, surgical, transcatheter, hybrid therapies and prognosis for patients with VSDs. It will focus exclusively on isolated VSDs excluding those associated with cyanotic CHDs such as tetralogy of Fallot, transposition of the great arteries, tricuspid atresia, and truncus arteriosus.

## 2. Classification of VSDs

The VSDs are most commonly classified on the basis of their size, number, and location in the ventricular septum [1, 2, 3, 4, 5, 6]. Sizes of VSDs can be categorized as large, medium, or small, and such assessment is helpful in categorizing patients into those that require intervention, and the defects may be single, paired, or numerous (multiple) [2, 3, 4]. Regarding location, they are categorized as perimembranous (situated in the subaortic area of the membranous ventricular septum), supracristal (found in the subpulmonary area of the conal septum), atrioventricular (AV) septal (located in the inlet septum), muscular (located in the muscular portion of the ventricular septum), and Gerbode (a defect involving the atrioventricular portion of the ventricular septum) [4, 5, 6, 7]. When multiple defects are observed in the muscular septum, they are described as the “Swiss cheese” VSDs.

## 3. Natural History of VSDs

An understanding of the natural history of VSDs is crucial for guiding the management of strategies of these conditions. It helps clinicians predict potential outcomes and decide the most appropriate interventions based on the defect’s progression and patient-specific factors. This knowledge forms the basis for evaluating the likelihood of spontaneous closure and other critical aspects addressed in subsequent sections.

### 3.1 Spontaneous Closure

Approximately 40% of VSDs close spontaneously, while 25% to 30% of VSDs may reduce in size sufficiently, obviating the need for therapeutic intervention [8, 9, 10, 11, 12]. The VSDs in the muscular septum are more likely to close than those in the membranous septum [10, 11]. While small VSDs demonstrate a higher closure rate than large defects (60% vs. 20%) [8, 9, 10, 11, 12], it should be noted that even large defects producing heart failure, or those needing banding of the pulmonary artery in early life, may undergo spontaneous closure [6]. Most VSDs close before 2 years of age, although the occurrence of spontaneous closure continues into adolescence and into adulthood [6, 8, 9, 10, 11]. The most common mechanism of closure involves the tricuspid valve leaflets juxtaposing against the VSD, a process also described as aneurysmal formation of the membranous ventricular septum [6].

### 3.2 Pulmonary Vascular Obstructive Disease

Development of pulmonary vascular obstructive disease (PVOD) is seen in nearly 10% of individuals with VSDs [6]. PVOD is believed to result from the pulmonary vascular bed being subjected to elevated pressure and increased blood flow [6]. Early identification and timely closure of the VSD, either through surgical or transcatheter methods, before the age of 18 months, is critical in reducing the prevalence of PVOD [6].

### 3.3 Development of Infundibular Stenosis

Infundibular obstruction develops in approximately 8% of individuals with VSDs [6], a phenomenon initially described by Gasul and his colleagues [6], which is described as Gasul’s transformation of the VSD. Early studies indicated that presence of right aortic arch and augmented angulation of the right ventricular outflow tract may result in Gasul’s transformation of the VSD [2, 3, 6]. Although the onset of infundibular obstruction ultimately needs surgical intervention, it indeed safeguards the pulmonary circuit and avoiding PVOD.

### 3.4 Aortic Insufficiency

Aortic insufficiency may develop in roughly 5% of individuals with VSDs. While the most common mechanism for spontaneous VSD closure involves tricuspid valve tissue, aortic valve leaflets may also contribute to VSD closure [6]. If that happens, aortic insufficiency develops. Such a phenomenon happens more frequently with supracristal VSDs than in other types. If aortic insufficiency is moderate to severe, surgical intervention to close the VSD and re-suspend the aortic valve leaflet.

## 4. Clinical Features

**Fig. 1.**
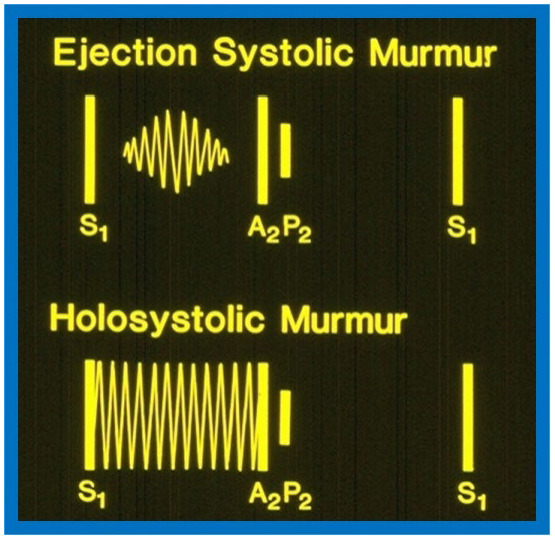
**Diagrams contrasting systolic murmurs on auscultation**. The diagram at the top illustrates an ejection systolic murmur, which begins shortly after the first heart sound (S_1_) and terminates just before the second heart sound, highlighting a gap between S_1_ and onset of the murmur. It also displays the two components of the second heart sound, namely, aortic (A_2_) and pulmonary (P_2_) components. In contrast, a holosystolic murmur shown at the bottom starts with S1, obscuring it, and continues through the systole as shown in the diagram, although it may terminate before reaching A_2_. Reproduced from Reference [3].

The VSD size dictates symptoms at presentation. Patients with small VSDs commonly do not have symptoms and are identified because a heart murmur heard on routine auscultation. Subjects with medium and large VSDs may have signs of congestive heart failure (CHF) such as rapid breathing, dyspnea, sweating, and inadequate weight gain or may have symptoms linked to bronchial obstruction and/or respiratory infection. Physical examination findings are also dependent on VSD size. Patients with small VSDs usually present with a loud holosystolic murmur best auscultated at the left lower sternal border, sometimes described as “maladie de Roger” (Fig. 1 bottom, Ref. [3]). Occasionally, the holosystolic murmur may be appreciated best at left mid and left upper sternal borders, dependent upon the path of the VSD jet [13]. In very small VSDs, while the murmur starts with first heart sound, may not persist during the entire systole; the shorter the murmur, the smaller is the defect.

Patients with medium and large VSDs often exhibit increased and hyperdynamic right and left ventricular (LV) impulses. A thrill is usually appreciated at the left lower sternal border. In cases of large VSDs, the second heart sound presents with a split and an accentuated pulmonary component [6]. Conversely, the second heart sound appears as a single sound in patients with PVOD. Additionally, clicks may occasionally be heard in patients with VSDs. A holosystolic murmur, ranging from grade II to V/VI, is usually auscultated best at the left lower sternal border, without radiation of the murmur to the other part of the precordium. However, this murmur may be appreciated extensively over the entire precordium, and does not vary with the respiratory cycle. There is no reliable correlation between the size of the defect and the intensity of the murmur. A mid diastolic murmur may be heard at apex and is produced by elevated blood flow through the mitral valve and typically suggests a pulmonary to systemic blood flow ratio (Qp:Qs) greater than 2:1.

## 5. Noninvasive Evaluation

**Fig. 2.**
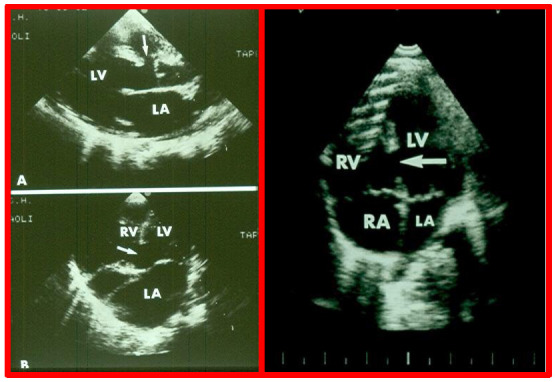
**Echocardiographic visualization of VSDs**. Selected video frames from a two-dimensional echocardiographic recording of the precordial long axis view (A) and the apical four chamber view (B). These frames highlight a VSD indicated by arrows, located immediately below the aorta (Ao) at the superior-most part of the interventricular septum (Left panel). An apical four-chamber view showing a muscular ventricular septal defect (arrow, Right panel). Note the difference in location between this and the ventricular septal defect shown in the left panel. LA, left atrium; LV, left ventricle; RA, right atrium; RV, right ventricle; VSD, ventricular septal defect. Reproduced from Reference [3].

**Fig. 3.**
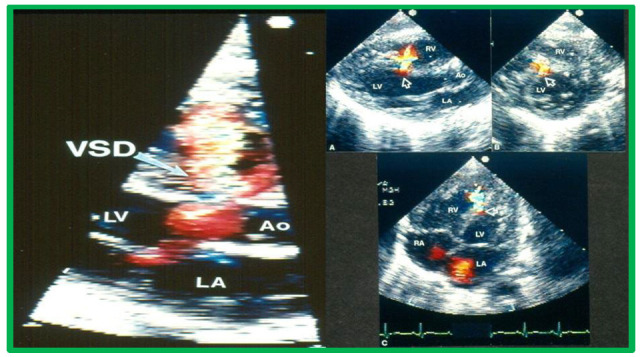
**Echocardiographic imaging of perimembranous VSDs**. Two-dimensional echocardiographic view of the ventricular septum in the long axis with color flow imaging (left panel), highlighting a perimembranous VSD and multiple views of the ventricular septum (arrows). Right panel (A–C) Multiple views of the ventricular septum demonstrating a left-to-right shunt through the VSD. Ao, aorta; LA, left atrium; LV, Left ventricle; RA, right atrium; RV, right ventricle; VSD, ventricular septal defect. Reproduced from Reference [3].

**Fig. 4.**
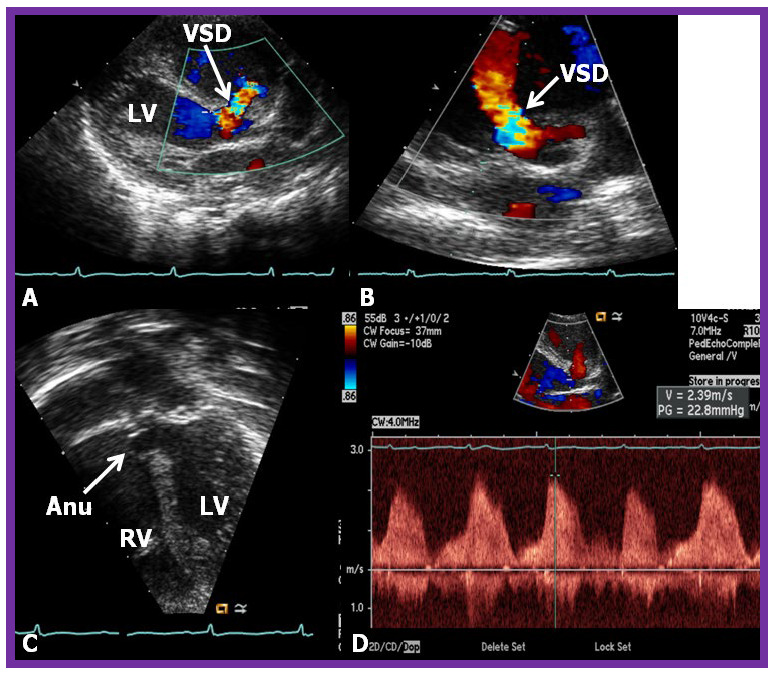
**Comprehensive echo-doppler analysis of a perimembranous VSD**. Echo-Doppler studies in parasternal long (A) and short (B) axis, along with apical four chamber projection (C), illustrating a perimembranous VSD with a shunt from the left ventricle (LV) to the right ventricle (RV). Color Doppler is used in (A,B), and continuous wave Doppler in (D) to illustrate the flow dynamics. The presence of an aneurysm (Anu) in (C) is suggestive of progression towards spontaneous closure. A Doppler flow velocity in excess of 2 meters/sec (D) suggests that the VSD is restrictive. VSD, ventricular septal defect. Reproduced from Reference [15].

**Fig. 5.**
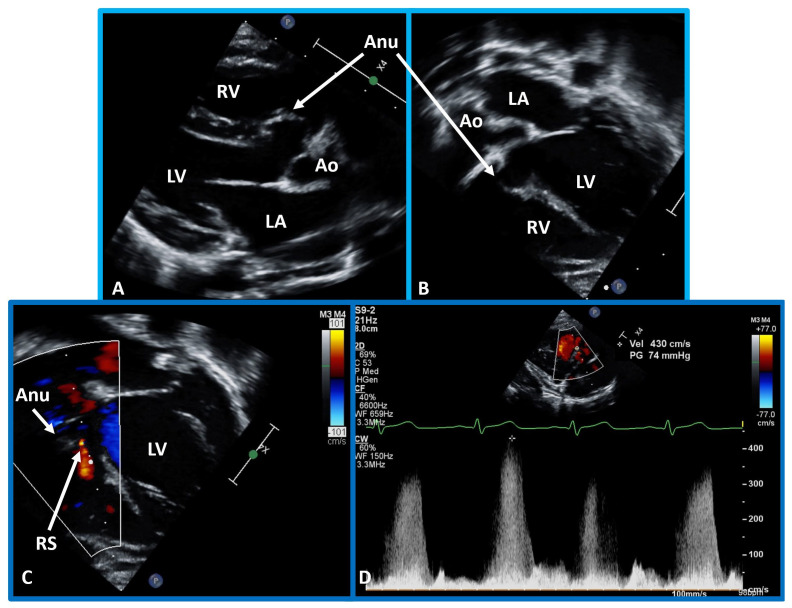
**Echo-Doppler visualization of a perimembranous VSD**. This figure shows Echo-Doppler recordings in parasternal long axis (A) and apical four chamber views (B,C) of the ventricular septum. These images illustrate a perimembranous VSD with a shunt from the LV to the RV by color (C) and continuous wave Doppler (D). The presence of an Anu in (A,B) is suggestive of progression to spontaneous closure. A high Doppler flow velocity (4.3 meters/sec, D) suggests that the VSD is restrictive, with normal pressures in the RV and pulmonary artery. Ao, aorta; LA, left atrium; RS, residual shunt; VSD, ventricular septal defect; LV, Left ventricle; RV, right ventricle.

**Fig. 6.**
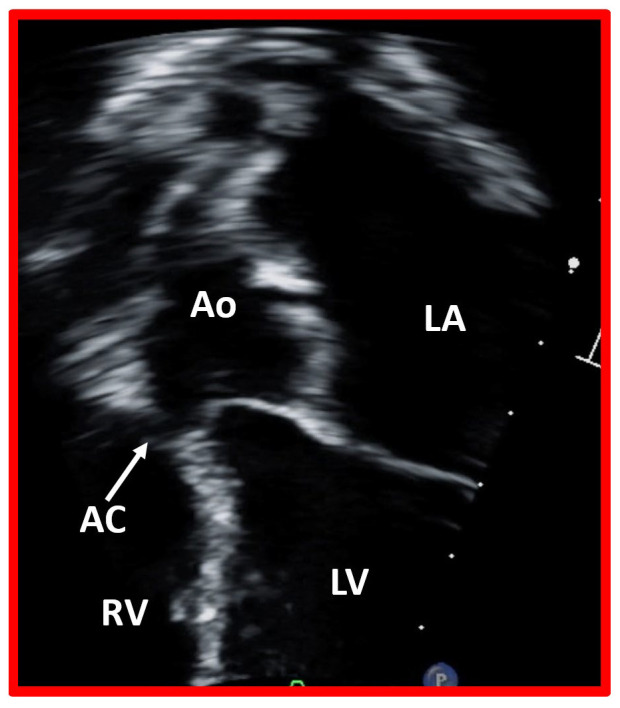
**Closure of ventricular septal defect by aortic cusp**. This image displays a modified apical four chamber view of the ventricular septum, illustrating the closure of a ventricular septal defect by the aortic cusp (AC). Ao, aorta, LA, left atrium; LV, left ventricle; RV, right ventricle.

While initial screening tests such chest x-ray and electrocardiogram (ECG) are helpful in the overall evaluation of VSDs, echocardiographic studies are crucial for confirming the diagnosis, assessing the degree of hemodynamic disturbance, and determining the need for medical, surgical or transcatheter intervention. On chest X-rays, cardiomegaly and increased pulmonary vascular markings typical findings in patients with medium to large VSDs, while the X-ray appears normal in cases of small VSDs. Similarly, the ECG typically shows normal results in small VSDs, but may reveal left atrial and left ventricular hypertrophy in moderate-sized VSDs, and biventricular hypertrophy in large VSDs. Right ventricular hypertrophy may be seen in patients who developed PVOD [2, 6]. The echocardiogram generally shows dilatation of the left atrium and left ventricle, which is largely proportional to the size of the VSD [14]. Two-dimensional imaging combined with color Doppler is effective in pinpointing the location of the VSD in the ventricular septum (Figs. 2,3, Ref. [3]). Echocardiographic studies can also demonstrate aneurysmal formations leading to spontaneous closure (as discussed in the Natural History section above) (Fig. 4, Ref. [15]; Figs. 5,6). It is essential to obtain multiple views of the VSD from different projections to adequately illustrate the closure process.

**Fig. 7.**
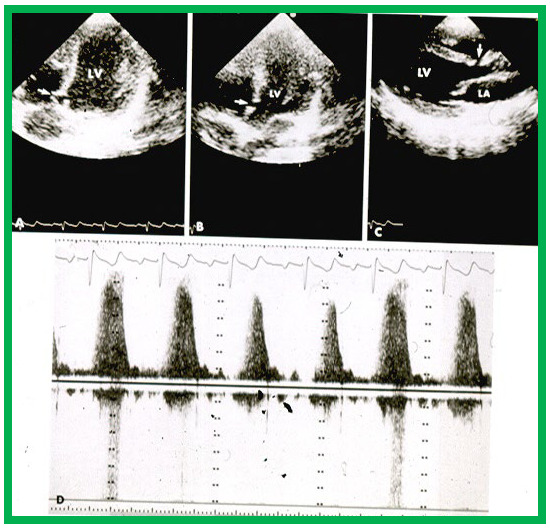
**High-velocity flow in a small VSD**. Echo-Doppler recording in the apical four-chamber (A,B) and parasternal long axis (C) projections demonstrate a small VSD (arrows in A–C), with increased flow velocity through the VSD, shown by continuous wave Doppler (D). Note the incomplete occlusion of the VSD (A) due to aneurismal formation. Left atrium (LA) and left ventricle (LV) are labeled. VSD, ventricular septal defect. Reproduced from Reference [16].

Shunting across the VSD can be shown by Doppler studies (Figs. 3,4). The magnitude of the peak Doppler flow velocity is inversely related to the size of the VSD; smaller defects produce higher Doppler velocities. Fig. 7 (Ref. [16]) illustrates a small VSD with high Doppler flow velocity.

The pulmonary arterial pressures can be estimated using the peak Doppler flow velocity through the VSD. The formula for estimating pulmonary artery peak pressure is:

PA peak pressure = peak arm blood pressure -4 ⁢ VVSD2

Here PA represents the pulmonary artery and V_VSD_ is the peak Doppler velocity across the VSD. Similarly, the peak pressure in the right ventricle can be calculated using the magnitude of the tricuspid regurgitation velocity:

$$\text{ RV peak pressure }={\text{ 4 TR}}^{2}-\text{4 RAP}$$

Where TR is peak tricuspid regurgitation velocity and RAP is estimated right atrial pressure (5 mmHg). These formulas are valuable for verifying the internal consistency of the Doppler methodology in estimating the size of the VSD. Generally, a higher estimated RV pressure suggests a larger dimension of the VSD.

PA, peak pressure, estimated peak pressure within the pulmonary artery; Peak arm blood pressure, the systolic blood pressure measured at the arm, typically during routine blood pressure monitoring; V_VSD_, peak Doppler velocity across the VSD, measured in meters per second and is used to estimate the pressure gradient across the VSD; RV peak pressure, estimated peak pressure within the right ventricle; TR, peak tricuspid regurgitation velocity, measured in meters per second, used to estimate the pressure gradient due to tricuspid valve regurgitation; RAP (right atrial pressure), estimated pressure within the right atrium, typically assumed to be 5 mmHg unless clinically indicated otherwise.

## 6. Computed Tomography (CT) and Magnetic Resonance Imaging (MRI)

While CT and MRI studies are useful in evaluating other types of CHDs, such studies are rarely necessary for the evaluation of VSDs.

## 7. Cardiac Catheterization & Cineangiography 

**Fig. 8.**
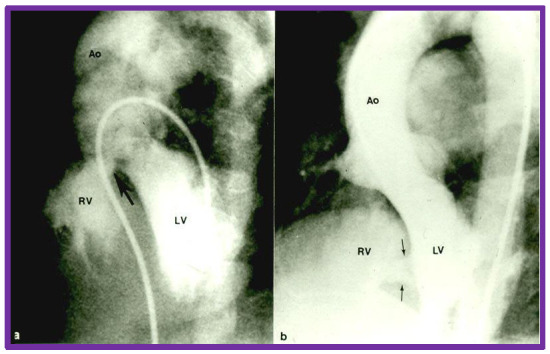
**Cine-angiographic depiction of VSDs**. Selected frames from left ventricular (LV) cine-angiograms in four chamber views showing ventricular septal defects. Panel (a) features a perimembranous VSD indicated by a large arrow, while panel (b) displays muscular VSDs, marked by two small arrows. Ao, aorta; RV, right ventricle; VSD, ventricular septal defect. Reproduced from Reference [3].

Historically, cardiac catheterization and selective cineangiography were essential in defining the characteristics of VSD prior to surgery. However, advancements in echo-Doppler methodologies have largely supplanted these methods by effectively identifying patients who would benefit from intervention. Consequently, cardiac catheterization is no longer routinely performed. It remains reserved for patients suspected of having high pulmonary vascular resistance and those needing vasoreactivity testing, and will not be reviewed in this paper. Selected cine frames illustrating perimembranous and muscular VSDs are shown in Fig. 8 (Ref. [3]).

## 8. Management of Ventricular Septal Defects

### 8.1 Indications for Intervention

The size of the VSD largely determines the need for intervention. Subjects with small VSDs typically do not require closure. It should be communicated to parents that the prognosis for these children is generally excellent, with infrequent follow-ups necessary until complete closure, and no activity restrictions should be stressed [6]. Some cardiologists recommend prophylaxis for subacute bacterial endocarditis [6].

However, if a small VSDs led to aortic valve cusp prolapse causing aortic insufficiency, surgical closure of the VCD along with resuspension of the aortic valve leaflets is advised [6]. For patients with moderate to large VSDs, intervention is necessary. Initial management should focus on addressing CHF, if present, followed by surgical or transcatheter closure, as appropriate [6]. Such closures should be accomplished between 6 to 12 months of age, and certainly prior to 18 months to prevent onset of PVOD. The time line may be expedited to approximately six months for infants with Down syndrome due to their increased risk of developing PVOD. Patients with moderate-sized VSDs with controlled CHF but with echocardiographic left heart dilatation should be observed for spontaneous resolution of the dilation. If no improvement is observed, closure of the VSD may be necessary. Conversely, patients with large VSDs who have developed severe elevation of pulmonary vascular resistance (PVR) and irreversible PVOD are not suitable candidates for VSD closure.

### 8.2 Caution

Given the straightforward nature of surgical procedures and the availability of transcatheter occlusion techniques for addressing small VSDs, there is notable inclination among surgeons and interventional cardiologists to proceed with VSD closures. However, since small VSDs frequently close spontaneously (as reviewed in the preceding section), pediatricians and pediatric cardiologists should exercise caution to avoid unnecessary interventions in such cases. Conversely, large VSDs with high pulmonary artery pressure tend to develop PVOD, a serious complication that must be prevented at all costs [6]. Pediatricians and pediatric cardiologists should prioritize timely intervention for the closure of these defects to mitigate the risk of developing PVOD.

### 8.3 Medical Management

Infants with moderate to large VSDs who show signs of CHF should be treated with anti-congestive medications such as afterload reducing agents (angiotensin-converting enzyme inhibitors including Captopril/Enalapril and others), diuretics (Furosemide and Aldactone), and digoxin [6]. Comprehensive care for these children should also involve optimizing nutrition, ensuring adequate levels of hemoglobin, and managing the respiratory problems. Additionally, most cardiologists recommend antibiotic prophylaxis prior to bacteremia producing procedures.

### 8.4 Surgical Management

Following the introduction of cardiopulmonary bypass procedures by Gibbon, Lillehei, and Kirklin in the 1950s to close atrial septal defects (ASDs) [17, 18, 19], surgical methods to address other CHDs, including VSDs, were soon established. The surgical procedure for VSD closure typically begins with the patient under general anesthesia, followed by a mid-sternal incision to open the chest. Cannulation of the aorta and vena cavae is undertaken to establish cardiopulmonary bypass. Most perimembranous VSDs are repaired with the use of a Dacron patch or similar prosthetic material via right atriotomy; sometimes tricuspid valve leaflet detachment may become necessary for better exposure of the VSD. VSDs located in the supracristal position are typically accessed and closed through the pulmonary valve. As mentioned in the section on “Indications for Intervention”, small VSDs partially closed by the aortic valve cusp, which lead to aortic insufficiency, also require surgical intervention to avoid further increase in aortic insufficiency [20]. Re-suspension of the prolapsed aortic valve leaflets or use of other valvuloplasty methods may become necessary in some patients with severe aortic valve prolapse [21, 22].

Large VSDs in the muscular septum, specifically the “Swiss cheese” type, are challenging to repair in infants. Consequently, initial banding of the pulmonary artery to manage CHF and decrease the pulmonary artery pressures was advocated in infants younger than three months of age. Subsequent closure of the VSD through an apical left or right ventriculotomy may be undertaken later during childhood [23, 24]. The pulmonary artery band is removed and the pulmonary artery anatomy is restored, if needed, to ensure that there is no residual narrowing at the site of the band. Interestingly, spontaneous closure of even very large muscular VSDs can occur following pulmonary artery banding [6] and in such situations, thoracotomy/stenotomy to remove the pulmonary artery becomes necessary. Given these potential outcomes, the use of an absorbable polydioxanone pulmonary artery band is advocated [6] similar to the approach for patients with tricuspid atresia with a large VSD [25, 26]. The absorbable band decreases pressure and blood flow in the pulmonary circuit at first and reduces symptoms related to CHF. After spontaneous closure of the VSD, the band is reabsorbed, and not requiring surgical removal. Despite these advantages, most pediatric cardiovascular surgeons remain hesitant to adopt the absorbable pulmonary artery band technique [6].

### 8.5 Results of Surgery

**Fig. 9.**
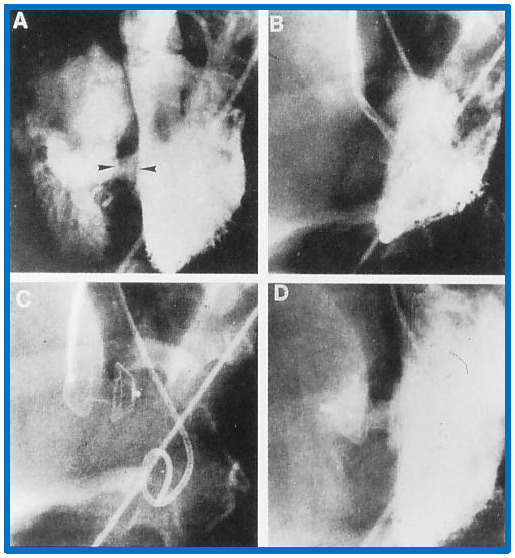
**Implantation and positioning of the Amplatzer muscular VSD occluder**. This figure shows selected frames from left ventricular (LV) cine-angiograms in left axial oblique views. Frame (A) illustrates the initial placement of Amplatzer muscular VSD Occluder (AMVO) indicated by the two arrow-heads. Frame (B) captures the position of the device during the procedure. Frame (C) details the position of the device immediately after placement. Frame (D) presents a post-release angiogram confirming the final placement of the device. Notation ‘c’ denotes the catheter used during the procedure. VSD, ventricular septal defect. Reproduced from Reference [3].

The safety of surgical repair of VSDs is well documented with a mortality rate of less than 1 to 3%. Historical data [27] and more recent studies [28, 29, 30, 31] both report excellent surgical outcomes with favorable long-term results. Occurrence of right bundle branch block is a frequent complication, but is generally well tolerated. Because of potential for long-term problems, some surgeons advocate use of different surgical techniques to reduce the prevalence of post-surgical right bundle branch block. Residual shunts, though rare (Fig. 9, Ref. [3]), often close spontaneously during follow-up [32]. The indications for reintervention in cases of residual defects are generally similar to those of native defects addressed above. Development of heart block and sinus node dysfunction have been reported, but are rare. Similarly, occasional pulmonary hypertension and infrequent increase in the degree of aortic insufficiency have also been reported. After VSD closure, there is typically a regression of the left atrial and left ventricular dimensions to normal, with restoration of normal left ventricular function parameters [33].

### 8.6 Percutaneous Closure

The historical aspects of transcatheter occlusion of VSD were reviewed in a prior publication [6] and will not be repeated in this paper. The majority of VSD occluding devices are double-disc devices, which require substantial septal rims for stable positioning. Consequently, they are useful in only occluding muscular defects and perimembranous VSDs with an adequate-sized aortic rim. Therefore, the more frequent perimembranous VSDs often lack this necessary rim, limiting the use of these devices. To circumvent this limitation, the devices were redesigned [34]; the distal end of the LV disc was lengthened, and the aortic end of the disc was shortened. Additionally, platinum marker was added to the lower end of the LV disc to aid interventionalist in recognizing the device orientation during implantation. This redesigned device was named Amplatzer Membranous VSD Occluder (St. Jude Medical, Inc.). This device was employed to occlude small- to medium-sized VSDs in FDA-approved studies [35] and other clinical trials [36, 37, 38, 39, 40, 41, 42, 43, 44], with outcomes generally considered satisfactory. However, the development of complete heart block, identified both at the time of the procedure and during follow-up, has raised significant concerns [45, 46, 47, 48, 49, 50, 51, 52]. This issue occurs more frequently with the Amplatzer Membranous VSD Occluder than with surgical methods, leading to critical evaluations of its use [53, 54]. A detailed analysis of the issues involved have been previously documented [54]. Because of the development of heart block in much higher percentage with the Amplatzer Membranous VSD Occluder than with surgery, device implantation technique will not be reviewed in this paper. However, muscular VSD occlusion will be reviewed in the next section.

### 8.7 Amplatzer Muscular VSD Occluder

The Amplatzer Muscular VSD Occluder is a specialized device designed for the transcatheter closure of muscular ventricular septal defects. Constructed from a nickel-titanium alloy known as Nitinol, which possesses shape-memory properties, this device features a unique double-disc structure linked by a central waist, facilitating effective and secure occlusion of VSDs [34, 55]. Specifically, the Nitinol wires have diameters of 0.004^′^^′^ or 0.005^′^^′^–titanium compound featuring shape-memory properties, and consisting of two equal-sized discs connected by a 7-mm-long waist, with each disc extending 4 mm beyond the waist. Dacron is incorporated within the discs to facilitate rapid occlusion, and the diameter of the waist determines the size of the device. Existing device sizes vary from 4 to 18 mm. Furthermore, it is possible to retrieve and reuse the device.

After securing the hemodynamic information, LV angiogram is first performed in long-axis oblique view with cranial angulation and straight lateral projections to pinpoint the VSD’s location and diameter. Historically, balloon sizing of the VSD was undertaken, but this procedure has been replaced by measurements from transesophageal echocardiography (TEE) and angiography to assess VSD size. The procedure involves passing a catheter (balloon wedge or right coronary artery) from the LV into the RV through the VSD, usually aided by a soft-tip guide wire, which is then exchanged for a longer one (Noodle wire, St. Jude Medical, Inc.). The tip of the guide wire is placed either in the superior vena cava or in the pulmonary artery. Then the tip of the wire is snared from the femoral vein, exteriorizing it, thus establishing an arterio-venous guide-wire loop. A suitable-sized delivery sheath is placed in the LV apex, and the guide wire and dilator are removed.

Alternatively, right internal jugular vein may be used to access the heart. In some cases, a retrograde arterial approach may be used. When venous access is chosen, a pigtail catheter is positioned retrogradely into the LV. For the procedure, an Amplatzer Muscular VSD Occluder, sized 1 to 2 mm larger than the VSD the diameter, is chosen for implantation. The device is securely attached to a delivery wire and carefully inserted into the delivery sheath, while ensuring that air bubbles are prevented from entering into the system. The device is pushed forward within the sheath, and the LV disc is delivered into the LV using both fluoroscopic and TEE monitoring.

The device’s placement is carefully adjusted to avoid interfering with the mitral valve function. The LV disc is withdrawn towards the ventricular septum with the aid of fluoroscopic and TEE control. If needed, LV angiogram is made to confirm the location of the device. The sheath is then gradually withdrawn, positioning the waist of the device within the VSD and aligning the RV disc with the RV aspect of the VSD. At this juncture, TEE and LV angiograms are performed to confirm satisfactory position of the device. Following this, the delivery wire is detached from the device and removed. A final LV angiogram and TEE are performed to ensure everything is properly set before the catheters and sheaths removed. The procedural steps, including these adjustments and checks, are illustrated in Fig. 9.

To ensure procedural safety and prevent complications during the implantation of intracardiac devices, several pharmacological measures are implemented. Initially, heparin (100 units/kg) is administered following insertion of catheters, with additional doses provided to maintain activated clotting times above 200 seconds. Concurrently, Ancef or a similar antibiotic (three doses eight hours apart) is administered. Aspirin or clopidogrel or both (anti-platelet therapy) may be given as per institutional practices for intracardiac device implantations.

The results of Amplatzer Muscular VSD Occluder [55, 56, 57, 58, 59, 60, 61, 62, 63, 64] were reviewed elsewhere [6] and will not be repeated in this review. Hybrid device delivery has also been used to address large VSDs [65, 66, 67, 68, 69, 70, 71, 72, 73, 74, 75, 76, 77, 78, 79, 80, 81, 82] and was reviewed in our prior publication [6] for the interested reader.

## 9. Summary and Conclusions

The diagnosis and management of VSDs was comprehensively reviewed in this paper. The classification of these defects is primarily based on their location within the ventricular septum. To get a greater understanding of the defect, natural history of the VSDs was reviewed which involves spontaneous closure, onset of pulmonary hypertension, development of infundibular obstruction, and evolution to aortic insufficiency. Echocardiography proves invaluable not only in diagnosing VSDs but also in quantifying their size and the appropriate determining indications for closure by surgical or transcatheter methods. Subjects with moderate- to large VSDs are generally candidates for treatment. Surgical closure of perimembranous, supracristal, and inlet VSDs is generally recommended. Patients with prolapsed aortic valve leaflets partially closing the VSDs are also candidates for surgical intervention. While transcatheter closure of perimembranous VSDs with Amplatzer Membranous VSD Occluder is technically feasible, it is not endorsed due to concerns regarding the potential for inducing complete heart block, a complication observed in a substantial number of patients. However, percutaneous and hybrid methods exist to close large muscular VSDs with Amplatzer Muscular VSD Occluder and generally yield favorable outcomes. In conclusion, while the majority of the treatment alternatives examined have demonstrated adequate results, it is crucial to exercise caution in the management of small defects to avoid unnecessary interventions. Conversely, the prompt and effective closure of large VSDs is critical to prevent the development of pulmonary vascular obstructive disease. This paper highlights the need for a balanced and patient-centric approach to the management of VSDs, emphasizing both safety and efficacy in therapeutic interventions.
